# Effects of Tidal Scenarios on the Methane Emission Dynamics in the Subtropical Tidal Marshes of the Min River Estuary in Southeast China

**DOI:** 10.3390/ijerph16152790

**Published:** 2019-08-05

**Authors:** Jiafang Huang, Min Luo, Yuxiu Liu, Yuxue Zhang, Ji Tan

**Affiliations:** 1 Institute of Geography, Fujian Normal University, Fuzhou 350007, China; 2 Environment and Resource College, Fuzhou University, Fuzhou 350116, China; 3 School of Geographical Sciences, Fujian Normal University, Fuzhou 35007, China

**Keywords:** tide, inundation, methane efflux, dissolved methane, tidal wetland, Min River Estuary

## Abstract

In order to accurately estimate the effects of tidal scenarios on the CH_4_ emission from tidal wetlands, we examined the CH_4_ effluxes, dissolved CH_4_ concentrations, and environmental factors (including in situ pH, Eh and electrical conductivity, porewater SO_4_^2−^, NO_3_^−^, and NH_4_^+^) during inundation and air-exposure periods in high- and low-tide seasons in the Min River Estuary in southeast China. By applying static and floating chambers, our results showed that the CH_4_ effluxes during the inundation periods were relatively constant and generally lower than those during the air-exposed periods in both seasons. When compared, the CH_4_ effluxes during the air-exposed periods were significantly higher in the high-tide season than those in the low-tide season. In contrast, CH_4_ effluxes during the inundation periods were significantly lower in the high-tide season than those in the low-tide season. During the inundation periods, dissolved CH_4_ concentrations were inversely proportional to in situ Eh. Under air-exposed conditions, CH_4_ effluxes were proportional to in situ pH in both seasons, while the dissolved CH_4_ concentrations were negatively correlated with the porewater SO_4_^2−^ concentrations in both seasons. Our results highlighted that CH_4_ effluxes were more dynamic between inundation and air-exposure periods compared to low- and high-tide seasons.

## 1. Introduction

Methane (CH_4_) is the second-most important radiatively active greenhouse gas and has a global warming potential 28 times greater than that of carbon dioxide (CO_2_) over a 100-year time span [1]. Despite a relatively small surface area, the volume of coastal CH_4_ effluxes was estimated to be quantitatively comparable to the CH_4_ uptake by the continental shelf [2]. Tidal wetlands, with an average net primary productivity of 930–7600 g C m^−2^·yr^−1^ [3], are important contributors of coastal CH_4_ effluxes [4]. Even though the quantification of CH_4_ effluxes from estuarine tidal wetlands is crucial when evaluating the global greenhouse budget, the data available for this purpose is extremely limited as estuarine tidal wetlands are periodically affected by fluctuating tidal hydrologic regimes [5].

The action of tides has a significant effect on CH_4_ effluxes through various physical, geochemical, and biological factors. First, tidal activity can induce changes in the hydrodynamic conditions, sediment deposition, and transport, as well as in the influxes and effluxes of mineral and organic compounds [6,7,8]. Second, tidal water is rich in oxygen (O_2_), sulfate (SO_4_^2−^), and high in salinity, which significantly affects methanogenesis and CH_4_ oxidation [9,10]. Third, the tides also influence various biological processes that have the potential to modify carbon cycling, such as microbial and plant respiration, photosynthesis, and carbon uptake [11,12].

Temporal variations in tidal activity can result in CH_4_ efflux dynamics [13]. Generally, studies on the impact of tidal activity on CH_4_ effluxes in estuarine tidal wetlands are based on a tidal, diel, or seasonal sampling timescale [5,14,15,16]. For instance, Hirota et al. [17] examined CH_4_ effluxes at 6 h intervals from 19 to 22 August along the sandy shore of Japan in mid-summer 2003; Rosentreter et al. [18] studied CH_4_ effluxes over 24-h periods in the wet and dry seasons in mangrove creeks along the north-eastern coast of Queensland, Australia, between March 2016 and March 2018; and Jacotot et al. [5] monitored CH_4_ emissions every three weeks throughout the high-tide period from the beginning of flooding to the end of the ebb in a Rhizophora mangrove forest (New Caledonia) from December 2016 to September 2017. In other research, Li et al. [19] continuously measured CH_4_ effluxes for two years using the eddy covariance technique in a subtropical salt marsh in eastern China in 2011 and 2012. A notable finding in previous studies was that CH_4_ effluxes exhibit a significant seasonal pattern with respect to tidal activity.

The coastal zone of China includes an 1800 km coastline that stretches across tropical, subtropical, and temperate zones and an extensive tidal wetland with an area of approximately 5.8 × 10^4^ km^2^ [20]. The tidal marshes of the Min River Estuary in the East China Sea cover ca. 21 km^2^ of subtropical coastal wetland. In the Min River Estuary, strong astronomical tides always occur between September and October. This is the combined result of the thermal expansion of seawater, the autumnal equinoctial spring tide, the western propagation of typhoons across the Pacific Ocean, and a reduction in river runoff [21]. During this period, the tide heights are significantly higher, the inundation periods are prolonged compared to other seasons, and the entire tidal flat is flooded most of the time. In contrast, during the winter and early spring, the tide height is much lower, the inundation period is significantly shorter, and the tide at times does not even reach the middle or upper regions of the tidal flats [22]. Previous studies have suggested that seasonal dynamics were apparent in the CH_4_ effluxes in the tidal wetlands of the Min River Estuary in the East China Sea, and that the highest CH_4_ effluxes occurred during the summer months (June to August) [22,23,24,25]. However, none of these investigated the influence of tidal activities on the dynamics of CH_4_ effluxes.

To explore this issue, in this study, the CH_4_ effluxes, dissolved CH_4_ concentrations, and environmental factors (including in situ pH, Eh and electrical conductivity (EC), porewater SO_4_^2−^, NO_3_^−^, and NH_4_^+^) were investigated in the tidal marshes of the Min River Estuary on an hourly basis from 26 September to 2 October 2011, and 23 March to 27 March 2012. The specific aims were (i) to explore the changes in the CH_4_ effluxes over seasonal and tidal scales, and (ii) to identify the environmental factors driving the dynamics of CH_4_ effluxes during the inundated and air-exposed periods.

## 2. Materials and Methods

### 2.1. Site and Sampling Period Description

**Figure 1:**
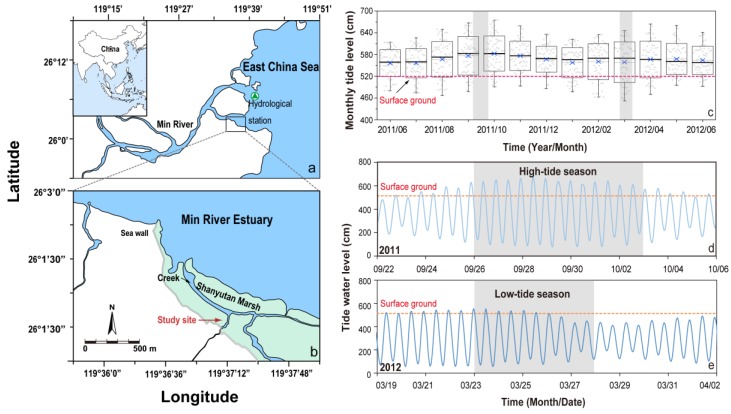
(**a**) Location of the study site in the Shanyutan brackish tidal marsh of the East China Sea, (**b**) the Min River Estuary, (**c**) patterns of tidewater level fluctuation (in cm) as assessed by the nearby hydrological station: monthly variation in the tide water level from June 2011 to June 2012, (**d**) daily tidewater level from 22 September to 6 October 2011, and (**e**) from 19 March to 2 April 2012. The shadow denotes the sampling period for the CH_4_ and porewater inventories. The red line denotes the surface of the ground at the study site. The tidewater level data was collected from the Chuanshi bathymetric benchmark (26°06′49″ N, 119°40′19″ E) of the China National Oceanic Bureau.

Located in the transitional zone between the middle and south subtropical zones, the Min River flows past vast tidal wetlands along the margins of its estuary in the East China Sea (Figure 1a,b). The tide of the Min River Estuary is a regular semi-diurnal tide with a range of 2.5–6.0 m, based on the data from the hydrological station (26°06′49″ N, 119°40′19″ E) of the China National Oceanic Bureau (http://ocean.cnss.com.cn/). The selected sampling site is located in a middle littoral zone within the Shanyutan brackish tidal marsh (26°1′–26°3′ N, 119°36′–119°38′ E). The site is primarily covered by the dominant species *Cyperus malaccensis* (also known as saltwater grass), a plant species of thin long sedge. The sediment is characterized by silty loam texture (i.e., 16–30% clay, 57–70% silt, and 7–12% sand).

### 2.2. Sample Collection and Handling

**Figure 2:**
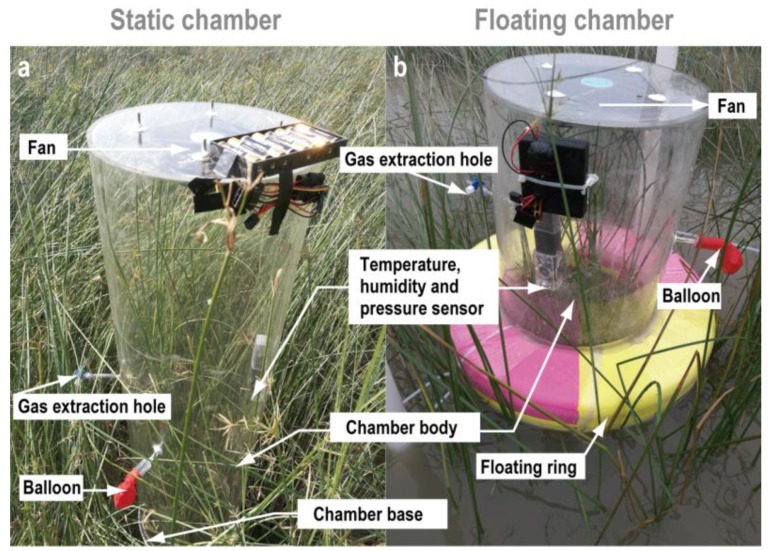
(**a**) Enclosed static chambers were used to measure the CH_4_ effluxes in the wetland at the sediment–air interface during the air-exposure period, and (**b**) floating chambers were used to measure the CH_4_ effluxes at the water–air interface during the tidal inundation period.

In this study, we defined the study period from 26 September to 2 October 2011 as the ‘high-tide season’, and that from 23 March to 27 March 2012 as the ‘low-tide season’. The sampling was conducted from 7:00 to 17:00 during the high-tide season and from 8:00 to 18:00 during the low-tide season. Enclosed static chambers were used to measure CH_4_ effluxes at the sediment–air interface during the air-exposed periods (Figure 2a), while floating chambers were used to measure the CH_4_ effluxes at the water–air interface during the inundation periods (Figure 2b). The enclosed static chamber was composed of two parts: a polyvinyl chloride bottom collar (diameter: 30 cm; depth: 25 cm), and a chamber body (diameter: 30 cm; height: 120 cm) with a temperature-humidity-pressure sensor and a small fan. The bottom collar was inserted into the wetland sediments, protruding 20 cm out of the sediment surface, approximately 7 d prior to the first sampling. The floating chambers (diameter: 30 cm; height: 40 cm) were made of transparent plexiglass; they were also provided with a temperature-humidity-pressure sensor and a small fan. Instead of a bottom collar, the floating chambers were set on swimming rings made of thick rubber. To prevent movement of the floating chambers during tidal fluctuation, the chambers were fixed by pipes inserted into the ground; therefore, the floating chambers could move up and down depending on the changes in tidewater height [22]. Triplicate enclosed static or floating chambers were separated from each other by approximately 10 m. Efflux measurements were conducted every 60 min during the air-exposed periods and every 45 min during the inundation periods. After the settling of the chamber bodies, all the edges were sealed with silicon and adhesive tape to ensure enough airtightness. Next, the fans were turned on and 30 mL of gas was immediately extracted with a syringe, and the operation was repeated thrice at 15 min intervals during each sampling period. Once a sampling cycle was completed, the chambers were removed and vented before the next sampling. Gas samples were collected in a vacuum-sealed 50 mL aluminum foil sampling bag (Delin Ltd., DaLian, China).

At each study location, triplicate rhizon samplers (20 mL) were installed on the sediment surface (10–15 cm) to collect porewater over time. Porewater was collected using in-situ rhizon samplers composed of a 0.15 μm hydrophilic porous polymer. All the gas and porewater were placed into sealed bags, and porewater subsamples were transported to the laboratory in an icebox (~4 °C).

### 2.3. Gas Flux Analysis and Estimation

Methane concentrations were determined using a gas chromatograph (GC-2014, Shimadzu, Kyoto, Japan) equipped with a thermal conductivity detector and a pulsed discharge detector. The column and detector temperatures were set at 60 °C. The carrier gas was high-purity helium, with a flow rate of 30 mL·min^−1^. Standard CH_4_ gas was produced by the Chinese Academy of Metrology (http://www.nim.ac.cn). The formula for calculating the gas flux is shown in Equation (1) [26,27]:

(1) $$F=\frac{1000}{V}\cdot \frac{\mathrm{dc}}{\mathrm{dt}}\cdot H\cdot \left(\frac{273}{273+T}\right),$$

where F is the CH_4_ efflux (μmol·m^−2^·h^−1^), V is the molar volume of gas (22.4 L·mol^−1^) under standard atmospheric pressure, dc/dt is the CH_4_ gas concentration per unit time change (μL·L^−1^·h^−1^) in the static chamber, H is the chamber height (m), and T is the sampling temperature.

### 2.4. Environmental Traits

Tide height (based on the ground surface) was recorded using a stainless steel water-level gauge every 15 min during the inundation periods. Tidewater salinity was examined by a SALT6+ salinity meter (EUTECH Instruments, Vernon Hills, IL, USA). Air temperature, precipitation, and humidity data were recovered from the U30-NRC HOBO weather station (Onset Computer Corporation, Bourne, MA, USA). In situ temperatures were measured using an IQ150 instrument (IQ Scientific Instruments, Carlsbad, CA, USA). The five-year tide water height and tide inundation frequency were obtained from Solinst 3001 (Solinst Canada Ltd., Georgetown, ON, Canada) data-loggers (±0.01 m) at 30-min intervals and were corrected by the China National Oceanic Bureau.

### 2.5. Sediment and Porewater Geochemistry

In situ electrical conductivity (EC) was measured with a Fieldscout 2265FS EC meter (Spectrum Technologies, Dallas, TX, USA). The in situ pH and Eh values were determined using an Orion 3-Star Plus pH/Eh benchtop meter (Thermo Fisher Scientific Inc., Waltham, MA, USA). In the laboratory, all porewater samples were handled under a nitrogen atmosphere. The porewater was filtered with 0.22 μm filters into 10 mL glass vials that were then capped with butyl rubber septa. The porewater subsamples were preserved at 4 °C and were analyzed within a month. Porewater SO_4_^2−^ concentrations were determined using ICS-2000 ion chromatography (Dionex, Sunnyvale, CA, USA). Porewater NO_3_^−^ and NH_4_^+^ concentrations were determined using a SAN++ flow injection system (Skalar Co., Breda, The Netherlands). The detection limits were 1 μM/8% for SO_4_^2−^, 10 μM/6% for NO_3_^−^, and 1 μM/4% for NH_4_^+^.

The dissolved CH_4_ concentration was determined using a headspace equilibration technique developed by Keller et al. [28] and Tong et al. [26]. Briefly, 15 mL of the first syringe porewater sample, obtained using rhizon samplers, was filtered through N_2_ preleached syringe filters (0.45 μm-pore) into a pre-vacuum glass scintillation vial, and was supplemented with 15 mL N_2_ to balance the difference in air pressure inside and outside the vial. For sterilization, 0.1 mL of saturated HgCl_2_ solution was injected into the vials. The bottles were then vigorously shaken for 30 s and placed on a rotary shaker (240 rpm, ambient temperature) for 0.5 h to ensure that all dissolved CH_4_ had escaped into the bottle headspace. The CH_4_ concentrations were measured using a GC2014 gas chromatograph with a flame-ionization detector (Shimadzu, Kyoto, Japan), typically within 2 h of collection. The measured CH_4_ concentrations were corrected for standard pressure and temperature using the ideal gas law and were multiplied by the headspace volume [26].

### 2.6. Data Analysis

All datasets were tested before the analysis of variance (ANOVA) to check that they meet the assumptions of homogeneity (the Brown and Forsythe test) and normality (the Shapiro–Wilk test). In cases where the data did not satisfy this assumption, the raw data were subjected to a log-transformation before further statistical analysis. A two-way analysis of variance (ANOVA) was used to determine whether the tide inundation periods (i.e., inundation and air-exposed periods), seasonality (i.e., low- and high-tide seasons), or an interaction between the two affected the soil CH_4_ effluxes or dissolved CH_4_ concentrations, as well as the environmental factors (in situ pH, Eh, and EC; porewater SO_4_^2^^−^, NO_3_^−^, and NH_4_^+^). The ANOVA tests were followed with an independent *t*-test to determine the difference between low- and high-tide seasons within each period, or between inundation and air-exposed periods within each season. Finally, nonlinear regressions between tide height and CH4 effluxes, between dissolved CH_4_ concentrations and in situ Eh during the inundation periods; linear regressions were used between the CH_4_ effluxes (or dissolved CH_4_ concentrations) and other environmental parameters during the air-exposed periods. All of the data in this study is presented in the form of the mean ± standard error (SE), unless otherwise indicated. All of the statistical analyses in this study were conducted using the SPSS Statistics 22.0 software program with a significance level of 0.05.

## 3. Results

Between June 2011 and June 2012, the monthly tide heights were the lowest in March and the highest between September and October (Figure 1c). Other hydrological and climatic condition data obtained during the two study periods are listed in Table 1. The mean inundation duration per day ranged from 2.2 h in the low-tide season to 8.1 h in the high-tide season (Table 1) and the monthly tide heights (with respect to the surface of the ground) were 123.7 cm and 16.4 cm in the high- and low-tide seasons, respectively (Table 1). The air temperature in the high-tide season (22.1 ± 0.1 °C) was relatively high compared with that in the low-tide season (17.5 ± 0.1 °C), and the humidity levels were comparable in the two seasons (Table 1). During the high-tide season, the salinity of the tide water was approximately 3.32 ppt, while during the low-tide season, the salinity of the tide water dropped to 0.73 ppt, possibly due to a heavy spring rainfall that diluted the salts and nutrients in the river or sea water.

### 3.1. Temporary Dynamics in CH_4_ Effluxes, Dissolved CH_4_ Concentrations, and Porewater Geochemistries

**Figure 3:**
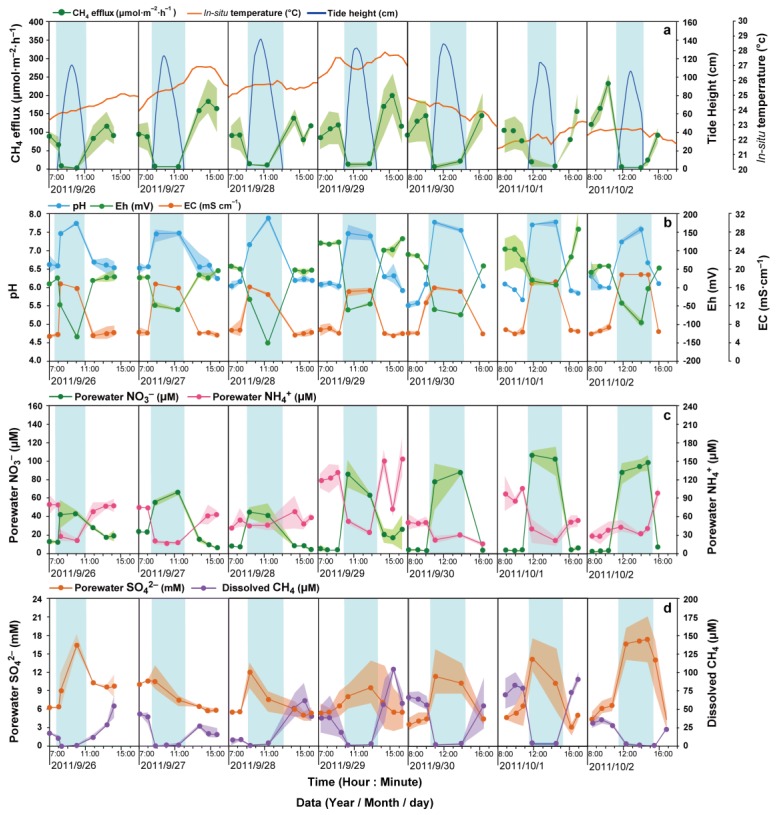
Dynamics of (**a**) the tide height (cm, water level above the ground surface), in situ temperature (°C), and CH_4_ effluxes (μmol·m^−2^·h^−1^); (**b**) EC (mS·cm^−1^), porewater pH, and Eh (mV); (**c**) NO_3_^−^ (μM) and NH_4_^+^ (μM); (**d**) SO_4_^2−^ (mM) and dissolved CH_4_ (μM) from tidal cycles in the period from 26 September 2011 to 2 October 2011 in the tidal wetlands of the Min River Estuary. The blue belt denotes the tidal inundation period and the shaded areas around the data denote the standard error.

**Figure 4:**
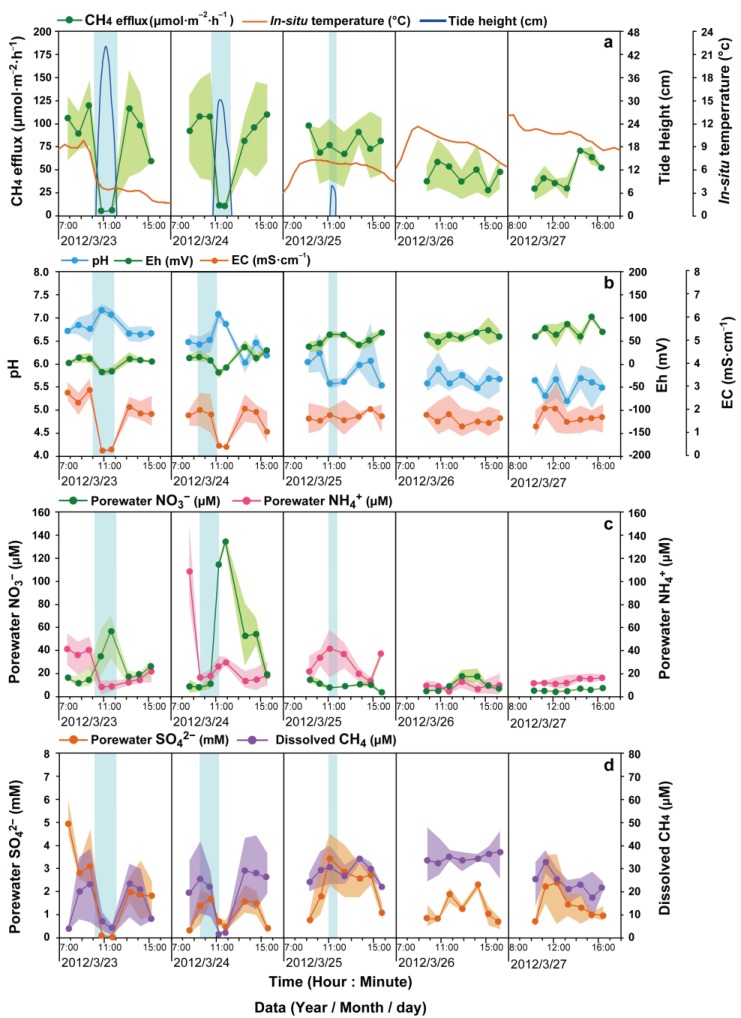
Dynamics of the (**a**) tide height (cm, water level above the ground surface), in situ temperature (°C), and CH_4_ effluxes (μmol·m^−^^2^·h^−^^1^); (**b**) EC (mS·cm^−^^1^), porewater pH, and Eh (mV); (**c**) NO_3_^−^ (μM) and NH_4_^+^ (μM); (**d**) SO_4_^2^^−^ (mM) and dissolved CH_4_ (μM) during tidal cycles between 23 March 2012 and 27 March 2012 in the sediments of the tidal wetland of the Min River Estuary. The blue belt denotes the tidal inundation period. The shaded areas around the data denote the standard error.

During the high-tide season, the sediments were inundated every day (Figure 3a). The tidal inundation periods first increased from 3.3 h to 4 h and then declined to 2.9 h, and were accompanied by a gradual increase in the maximal tide height from 113 cm to 140 cm, followed by a decline to 106 cm (Figure 3a). During the low-tide season, the tidal inundation periods gradually decreased from 1.9 h to 0.6 h, and the maximal inundation height gradually decreased from 44 cm to 8 cm (Figure 4a). The tide did not reach the study site in the last two days of the low-tide season (Figure 4a).

**Figure 5:**
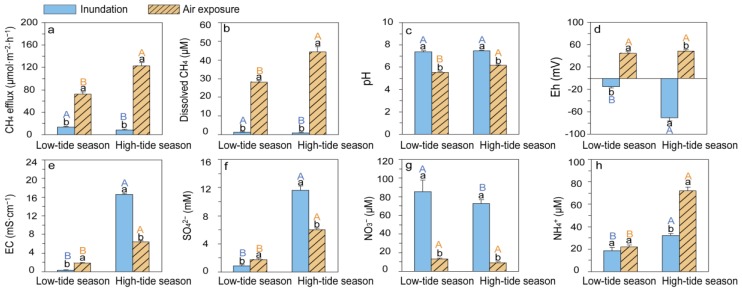
(**a**) CH_4_ effluxes (μmol·m^−2^·h^−1^), (**b**) dissolved CH_4_ (μM), (**c**) in situ pH, (**d**) Eh (mV), (**e**) EC (mS·cm^−1^), (**f**) porewater SO_4_^2^^−^ (mM), (**g**) porewater NO_3_^−^ (μM), and (**h**) NH_4_^+^ (μM) in the tidal wetlands of the Min River Estuary. The solid blue and shadowed orange bars denote inventories made during inundation and air exposure periods, respectively. The capital letters in colors indicate significant differences (*p* < 0.05 after an independent *t*-test) between the low- and high-tide seasons. The small letters in black indicate significant differences (*p* < 0.05 after the independent *t*-test) between the inundation and air-exposed periods.

The CH_4_ effluxes (given in μmol·m^−2^·h^−1^, Figure 3a and Figure 4a) ranged from 1.0 to 248.3. During both the high- and low-tide seasons, the CH_4_ effluxes during the inundation periods were much lower than those during the air-exposed periods (Figure 5a) and the dissolved CH_4_ concentrations (given in μM, Figure 3d and Figure 4d) generally followed a trend similar to that of the CH_4_ effluxes (r = 0.47, *p* < 0.001, n = 258), i.e., they were low during the inundation periods (<5 μM) and much higher during the air-exposed periods (8.3 to 103.29 μM) in both seasons (Figure 3d and Figure 4d). Regarding the differences between the two seasons, the CH_4_ effluxes and dissolved CH_4_ concentrations from air-exposed sediments were significantly higher during the high-tide season compared with the low-tide season, while the CH_4_ effluxes and concentrations of dissolved CH_4_ during the inundation periods were significantly lower during the high-tide season compared with the low-tide season (Figure 5a,b).

### 3.2. Difference in CH_4_ Effluxes, Dissolved CH_4_ Concentrations, and Porewater Geochemistries

The results of a two-way ANOVA showed that both the tide inundation and seasonality affected the CH_4_ effluxes, dissolved CH_4_ concentrations, in situ pH, Eh and EC, concentrations of porewater SO_4_^2−^, NO_3_^−^, and NH_4_^+^ (Table 2).

The in situ pH was slightly acidic during the air-exposed periods (low-tide season: 5.55; high-tide season: 6.28) and turned neutral (low-tide season: 7.05; high-tide season: 7.39) during the inundation periods in both seasons (Figure 5c). Moreover, in situ Eh was negative during the inundation periods (low-tide season: −15.26; high-tide season: −70.62) and became positive (low-tide season: 48.91; high-tide season: 54.82) during the air-exposed periods in both seasons (Figure 5d).

During the low-tide season, the in situ EC (mS·cm^−^^1^; Figure 5e) and SO_4_^2^^−^ concentrations (mM; Figure 5f) were much lower during the inundation periods (EC = 0.34; SO_4_^2^^−^ = 0.86) than during the air-exposed periods (EC = 1.77; SO_4_^2^^−^ = 1.77). In contrast, during the high-tide season, the in situ EC and porewater SO_4_^2^^−^ were much higher during the inundation periods (EC = 16.53; SO_4_^2^^−^ = 11.63) than during the air-exposed periods (EC = 6.43; SO_4_^2^^−^ = 6.01).

During inundation, porewater NO_3_^−^ (low-tide season = 85.24; high-tide season = 72.84) was much higher than that during the air-exposed periods (low-tide season = 13.06; high-tide season = 9.38) in both seasons (Figure 5g). In addition, both inundated and air-exposed porewater NH_4_^+^ were relatively less abundant in the low-tide season (inundation = 17.75; air-exposed = 20.65) compared with the high-tide season (inundation = 32.19; air-exposed = 72.15; Figure 5h).

### 3.3. Relation between CH_4_ Effluxes or Dissolved CH_4_ Concentrations and Other Environmental Factors

**Figure 6:**
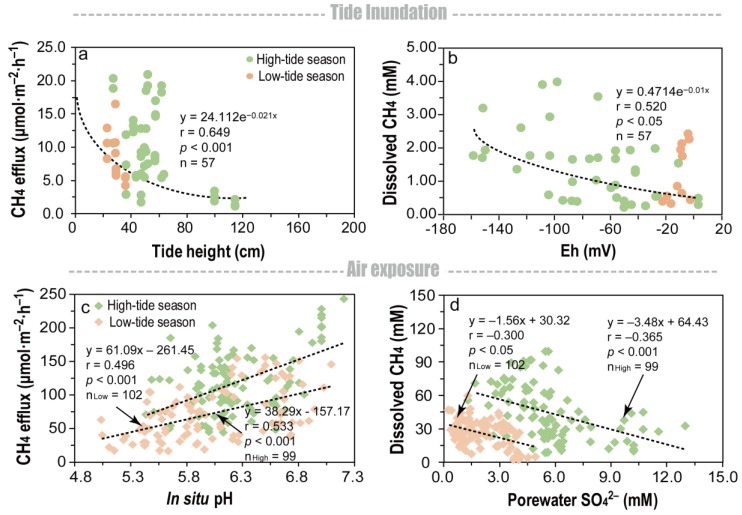
(**a**) Exponential regression correlation between the tide height and CH_4_ effluxes; (**b**) exponential regression correlation between dissolved CH_4_ concentrations and Eh, during inundation conditions; (**c**) linear regression correlation between porewater pH and CH_4_ effluxes; and (**d**) linear regression correlation between dissolved CH_4_ and porewater SO_4_^2^^−^ during air-exposed conditions in high- and low-tide seasons, respectively. ‘r’ and ‘p’ refer to regression coefficient and significance level of the regression model, respectively. “n”, “n_high_”, and “n_low_” refer to the data in both high- and low-tide seasons, high-tide seasons, and low-tide seasons, respectively.

During the inundation periods, CH_4_ effluxes and dissolved CH_4_ concentrations exponentially declined with an increase in tide heights and Eh, respectively (Figure 6a,b). During the air-exposed periods, CH_4_ effluxes linearly increased with a promotion in in situ pH (Figure 6c), and dissolved CH_4_ concentrations declined with an increase in porewater SO_4_^2^^−^ concentrations (Figure 6d) in the high- and low-tide seasons, respectively.

## 4. Discussion

The aim of this study was to explore the tidal scenario effects on CH_4_ effluxes between two seasons and identify the environmental factors driving the dynamics of CH_4_ effluxes during inundated and air-exposed periods in high- and low-tide seasons. Although maximal tide heights changed every day, the profiles of CH_4_ effluxes and dissolved CH_4_ concentrations over a tidal cycle (inundation to air-exposure to inundation) were relatively constant throughout the high-tide season (Figure 3a). By contrast, the profiles were much more heterogeneous during the low-tide season (Figure 4a), due to the fact that the sediments were totally dried up in some periods of the tidal cycle. Our results showed that both tide inundation and seasonality showed a significant influence on CH_4_ effluxes and dissolved CH_4_ concentrations, and that tide inundation had a more pronounced impact on CH_4_ dynamics compared with seasonal change (Figure 5).

### 4.1. CH_4_ Effluxes and Dissolved CH_4_ Concentrations during the Inundation Periods between Two Seasons

The current study identified an inverse relationship between the fluctuations in the CH_4_ effluxes and the tide progression within a tidal cycle (Figure 3 and Figure 4), and the tidewater was found to inhibit the CH_4_ effluxes during each tidal cycle in both seasons (Figure 3 and Figure 4). Then, as the sediments became exposed to air after the tidal ebb, the CH_4_ effluxes rebounded to almost their original levels (Figure 3 and Figure 4). Similar patterns have been reported in many coastal ecosystems that are frequently inundated by tidewater [7,29,30]. The cycle of inhibition and rebound occurred over a short period of time. Moreover, it was found that during periods of inundation, there was a negative exponential relationship between the magnitude of the CH_4_ effluxes and the height of the tide water in both seasons (Figure 6a). A likely explanation of the inhibition is that tidewater pressure prevents diffusion of the CH_4_ when the pore spaces in the sediment become filled with water [5,22,26,31,32].

The variation in the profiles of dissolved CH_4_ were consistent with those of the CH_4_ effluxes (Figure 3 and Figure 4). Since the porewater sampling depth was 10–15 cm in this study, fine-grained sediments (in the form of clay and silt) likely prevented porewater exchange with the air or overlying tide water [33,34,35,36]. Dissolved CH_4_ at this depth can thus be seen to a certain extent as a proxy of methanogenesis. Consistent with previous studies [29,37,38], the decreasing redox potential favored the accumulation of dissolved CH_4_ during the inundation periods (Figure 6b) as the favorable redox potentials for methanogenic activity are in the range of −300 to −150 mV [10,39,40,41]. A reduced and anoxic environment may lead to an enrichment in methanogens, indicating that the dissolved CH_4_ was indirectly affected by the redox conditions during the inundation periods.

### 4.2. CH_4_ Effluxes and Dissolved CH_4_ Concentrations during the Air-Exposed Periods between the Two Seasons

Higher CH_4_ effluxes were found during the air-exposed periods in the high-tide season compared with the low-tide season (Figure 5a,b), with there being many possible explanations for this. Most importantly, the results of previous studies suggest that CH_4_ effluxes are sensitive to temperature and plant growth characteristics (e.g., biomasses, primary productivities, and root exudes) [22,23,24,25], and that higher temperatures may stimulate relatively higher methanogenic activity in sediments during high-tide seasons compared with low-tide seasons [19,42,43]. In addition, as the plant biomass was more abundant in the high-tide seasons than the low-tide seasons (Table 1), this may provide more carbon-rich substrate to the methanogens [8,44].

The design of our experiment made it difficult to assess the role of the temperature, plant, or sediment and porewater geochemistries in the seasonal differences noted in the CH_4_ effluxes in this study. However, we found that the porewater SO_4_^2^^−^ and in situ pH had a definite influence on the CH_4_ dynamics during the air-exposed periods within each season. During the air-exposed periods, CH_4_ effluxes linearly increased with the sediment pH in the high- and low-tide seasons (Figure 6c). For each increase of one pH unit, the CH_4_ effluxes significantly increased to approximately 61 and 38 mmol m^−2^ d^−1^ in the high- and low-tide seasons, respectively (Figure 6c). The most likely mechanisms by which the pH can regulate CH_4_ production are related to acetoclastic methanogenesis [45]. For many mineral wetlands, the acetoclastic methanogenesis pathway has been reported to dominate CH_4_ production [46], and slightly acidified sediments have been found to reduce CH_4_ production in flooded rice [47], peatland [45], and tidal marsh soils [48].

Sulfates are important factors that limit methanogenesis in tidal marsh sediments [49], and the results of previous studies have found an inhibitory effect of SO_4_^2^^−^ on the concentrations of dissolved CH_4_ [50,51,52]. In the current study, a negative relationship was observed between the dissolved CH_4_ during the air-exposed periods and the porewater SO_4_^2^^−^ in both the high- and low-tide seasons (Figure 6d). There are two possible explanations for this negative correlation: (i) microbial sulfate reduction is thermodynamically favored over CH_4_ production in the competition for organic substrates as the metabolic process of methanogenesis yields low energy [53]; (ii) sulfate reduction is an important anaerobic CH_4_ oxidation pathway in marine and coastal sediments [54,55].

### 4.3. Reassessment of Tide-Independent CH_4_ Effluxes

In previous studies, CH_4_ effluxes were typically assessed based on data from only the air-exposed periods; however, that approach may considerably overestimate the actual CH_4_ efflux budget in the tidal marsh sediments. In this study, the tide height and inundation frequencies varied seasonally; however, we found that the CH_4_ effluxes exponentially decreased with tide height in the inundation periods (Figure 6a). We, therefore, could estimate the seasonal CH_4_ effluxes during the inundation periods using the following equation:

CH_4_ efflux_inundation_ = 24.112 e^−0.021TH^, (2)

where CH_4_ efflux_inundation_ indicates the seasonal CH_4_ effluxes during the inundation periods, and TH refers to the seasonal mean tide height.

The tide height and inundation frequencies varied seasonally. With long-term observations of tide hydrological data (tide height) and using Equation (2), we could also estimate the seasonal CH_4_ efflux_inundation_. In addition, as shown previously [25], we had five years of observational data of seasonal CH_4_ effluxes (only from air-exposure periods). Combined with this and other previous studies, we estimated the adjusted annual CH_4_ effluxes (including both inundation and air-exposed periods) as follows:

CH_4_ efflux_adjust_ = CH_4_ efflux_inundation_ × IR% + CH_4_ efflux_air-exposed_ × (1 − IR%), (3)

where CH_4_ efflux_adjust_ refers to the adjusted annual CH_4_ effluxes (containing both inundation and air-exposed periods), CH_4_ efflux_air-exposed_ refers to the seasonal CH_4_ effluxes during the air-exposed periods, and IR% indicates the ratio of seasonal inundation duration per month.

The results (Table 3) suggested that the adjusted seasonal CH_4_ effluxes amounted to only 55–91% of the original CH_4_ effluxes that were based on data obtained during the air-exposed periods. Moreover, the tide-dependent annual CH_4_ efflux budget was only 76% of the original annual CH_4_ efflux budget.

## 5. Conclusions

In this work, we examined the CH_4_ dynamics under inundation and air-exposed periods across the high- and low-tide seasons. Our results showed that both tide inundation and seasonality showed a significant influence on CH_4_ effluxes and dissolved CH_4_ concentrations, and tide inundation had a more pronounced impact on CH_4_ dynamics compared to seasonal change. The CH_4_ effluxes during the air-exposed periods (17.6 to 248.3 mmol·m^−2^·h^−1^) were highly variable and generally higher than those during inundation periods (1.0 to 22.0 mmol·m^−2^·h^−1^) within a tidal cycle in both seasons. Most notably, between the two seasons, the CH_4_ effluxes and dissolved CH_4_ concentrations from the air-exposed sediments were significantly higher during the high-tide season compared with the low-tide season, while the CH_4_ effluxes and dissolved CH_4_ concentrations from the inundated sediments were significantly lower during the high-tide season compared with the low-tide season. When the tide inundation frequency was taken into account, the adjusted annual budget of CH_4_ effluxes amounted to 76% of the CH_4_ effluxes in the air-exposed data.

To identify the effects of tidal scenarios on the CH_4_ efflux dynamics in the tidal marshes, we chose the two seasons that had radically different hydrological conditions within a year. We found a significant relationship between tidal height and CH_4_ efflux. However, the lack of long-term observational data limits the potential applications of this relationship. To more accurately estimate the CH_4_ budget in tidal wetlands, future sampling strategies should include additional measurements of the CH_4_ effluxes during both the inundation and air-exposed periods in the context of different tidal scenarios. However, tide inundation and other environmental factors, such as temperature, light, precipitation, vegetation and bioturbation, ground water table, and rise in sea-level, all vary throughout the decades, years, seasons, days, or tidal periods and, thus, intensive studies over longer time scales are needed. Even if real-time monitoring is not available, a rough first estimate of the inundated CH_4_ effluxes would be possible using information on tidal heights and inundation periods.

## Figures and Tables

**Table 1:** Hydrological and climatic conditions (mean ± SE) of high- and low-tide seasons in the tidal wetlands of the Min River Estuary, East China Sea.

	High-Tide Season	Low-Tide Season
Sampling period	26 September to 2 October 2011	23 to 27 March 2012
Mean air temperature (°C)	22.1 ± 0.1	17.5 ± 0.1
Mean humidity (%)	90.5 ± 0.3	99.9 ± 0.1
Mean highest tidal height (cm) *	123.7 ± 7.4	16.4 ± 11.1
Mean inundation duration per day (h)	8.1 ± 5.1	2.2 ± 1.4
Mean tidewater salinity (ppt)	3.32 ± 0.15	0.73 ± 0.01

Note: * Tidal height was calculated based on the surface ground of study site.

**Table 2:** Summary of a two-way ANOVA on the effects of tide inundation, seasonality, and their interaction on the methane effluxes and dissolved CH_4_; the in situ pH, Eh, and EC; and the porewater SO_4_^2^^−^, NO_3_^−^, and NH_4_^+^ in the tidal marsh of the Min River Estuary, East China Sea.

Source	Tide Inundation		Season		Inundation × Season	
	*F* Value	*p* Value	*F* Value	*p* Value	*F* Value	*p* Value
CH_4_ efflux	140.173	<0.001	12.842	<0.001	10.340	0.001
Dissolved CH_4_	116.379	<0.001	6.695	0.010	6.291	0.013
In situ pH	275.113	<0.001	19.358	<0.001	3.151	0.077
In situ Eh	361.573	<0.001	29.322	<0.001	39.922	<0.001
In situ EC	502.431	<0.001	2932.265	<0.001	903.409	<0.001
Porewater SO_4_^2^^−^	31.943	<0.001	321.832	<0.001	60.722	<0.001
Porewater NO_3_^−^	525.225	<0.001	7.388	0.007	2.169	0.142
Porewater NH_4_^+^	21.983	<0.001	47.971	<0.001	15.693	<0.001

**Table 3:** Estimation of seasonal methane effluxes using tidal height and inundation frequency.

	Spring	Summer	Autumn	Winter
TH (cm)	16.4 ± 11.1	65.7 ± 13.4	123.7 ± 7.4	26.8 ± 17.7
IR (%)	13.0 ± 5.7	22.0 ± 6.8	33.6 ± 2.1	18.8 ± 7.6
CH_4_ efflux_air-exposed_ (μmol·m^−^^2^·h^−^^1^)	60.8	416.3	188.6	39.5
CH_4_ efflux_inundation_ (μmol·m^−^^2^·h^−^^1^)	16.4	6.1	1.8	13.7
CH_4_ efflux_adjust_ (μmol·m^−^^2^·h^−^^1^)	55.0	326.0	125.8	21.9

Notes: TH refers to the seasonal mean tide height (Equation (2)); CH_4_ efflux_inundation_ indicates the seasonal CH_4_ effluxes during the inundation periods (Equation (2)); IR% indicates the ratios of seasonal inundation duration per month (Equation (3)); CH_4_ efflux_air-exposed_ refers to the seasonal CH_4_ effluxes during the inundation periods (Equation (3)); CH_4_ efflux_adjust_ refers to the adjusted annual CH_4_ effluxes (including both inundation and air-exposure periods; Equation (3)).
